# Contamination with HIV antibody may be responsible for false positive results in specimens tested on automated platforms running HIV 4th generation assays in a region of high HIV prevalence

**DOI:** 10.1371/journal.pone.0182167

**Published:** 2017-07-31

**Authors:** Diana Ruth Hardie, Stephen N. Korsman, Nei-Yuan Hsiao, Molefi Daniel Morobadi, Sabeehah Vawda, Dominique Goedhals

**Affiliations:** 1 Division of Virology, Department of Pathology, University of Cape Town, Cape Town, South Africa; 2 National Health Laboratory Service, South Africa; 3 Department of Medical Microbiology and Virology, University of the Free State, Bloemfontein, South Africa; "INSERM", FRANCE

## Abstract

**Introduction:**

In South Africa where the prevalence of HIV infection is very high, 4^th^ generation HIV antibody/p24 antigen combo immunoassays are the tests of choice for laboratory based screening. Testing is usually performed in clinical pathology laboratories on automated analysers. To investigate the cause of false positive results on 4^th^ generation HIV testing platforms in public sector laboratories, the performance of two automated platforms was compared in a clinical pathology setting, firstly on routine diagnostic specimens and secondly on known sero-negative samples.

**Methods:**

Firstly, 1181 routine diagnostic specimens were sequentially tested on Siemens and Roche automated 4^th^ generation platforms. HIV viral load, western blot and follow up testing were used to determine the true status of inconclusive specimens. Subsequently, known HIV seronegative samples from a single donor were repeatedly tested on both platforms and an analyser was tested for surface contamination with HIV positive serum to identify how suspected specimen contamination could be occurring.

**Results:**

Serial testing of diagnostic specimens yielded 163 weakly positive or discordant results. Only 3 of 163 were conclusively shown to indicate true HIV infection. Specimen contamination with HIV antibody was suspected, based on the following evidence: the proportion of positive specimens increased on repeated passage through the analysers; viral loads were low or undetectable and western blots negative or indeterminate on problem specimens; screen negative, 2^nd^ test positive specimens tested positive when reanalysed on the screening assay; follow up specimens (where available) were negative. Similarly, an increasing number of known negative specimens became (repeatedly) sero-positive on serial passage through one of the analysers. Internal and external analyser surfaces were contaminated with HIV serum, evidence that sample splashes occur during testing.

**Conclusions:**

Due to the extreme sensitivity of these assays, contamination with minute amounts of HIV antibody can cause a negative sample to test positive. Better contamination control measures are needed on analysers used in clinical pathology environments, especially in regions where HIV sero-prevalence is high.

## Introduction

South Africa has the largest HIV epidemic in the world, with an estimated 5.7 million infections [1]. In the public sector, HIV testing is usually performed in clinics using rapid test devices. However, ensuring a uniform quality of testing at point of care can be difficult [2][3]. In hospitalised patients, blood is sent to a laboratory for testing. Laboratory screening involves the use of 4^th^ generation antibody/p24 antigen combo immunoassays. Initially reactive specimens are tested with a second 4^th^ generation immunoassay. Patients who are reactive in both are considered HIV-infected, in line with World Health Organisation recommendations [4]. Public health laboratories routinely request a second specimen in newly diagnosed patients, but frequently clinicians fail to send follow up samples.

4^th^ generation HIV immunoassays have superior sensitivity and specificity to 3^rd^ generation rapid tests currently in use in clinics in South Africa [5]. They are designed to detect infection as early as possible after infection and specimens containing minute amounts of HIV antibody can test positive. In the interest of logistic efficiency, serology is commonly performed on automated chemistry analysers in consolidated clinical pathology laboratories. In this setting, weakly positive HIV serology is commonly encountered. Use of confirmatory assays seldom resolves the problem and the HIV diagnosis remains uncertain, requiring follow up serology and molecular testing to resolve. Factors responsible for false reactivity in HIV immunoassays include: presence of cross-reacting antibody [6], inadequate centrifugation, haemolysis or lipaemia in the specimen. Inadvertent contamination of negative specimens with HIV positive serum during the analytical process may also be responsible.

Following the recognition of an unacceptably high rate of false positive results on several 4^th^ generation HIV testing platforms, a field study was done to compare the performance of two platforms and identify the cause of false positive results.

## Methods

### Design

Ethics approval for this study was obtained from the University of Cape Town Research Ethics Committee (reference number 632/2016).

**Fig 1:**
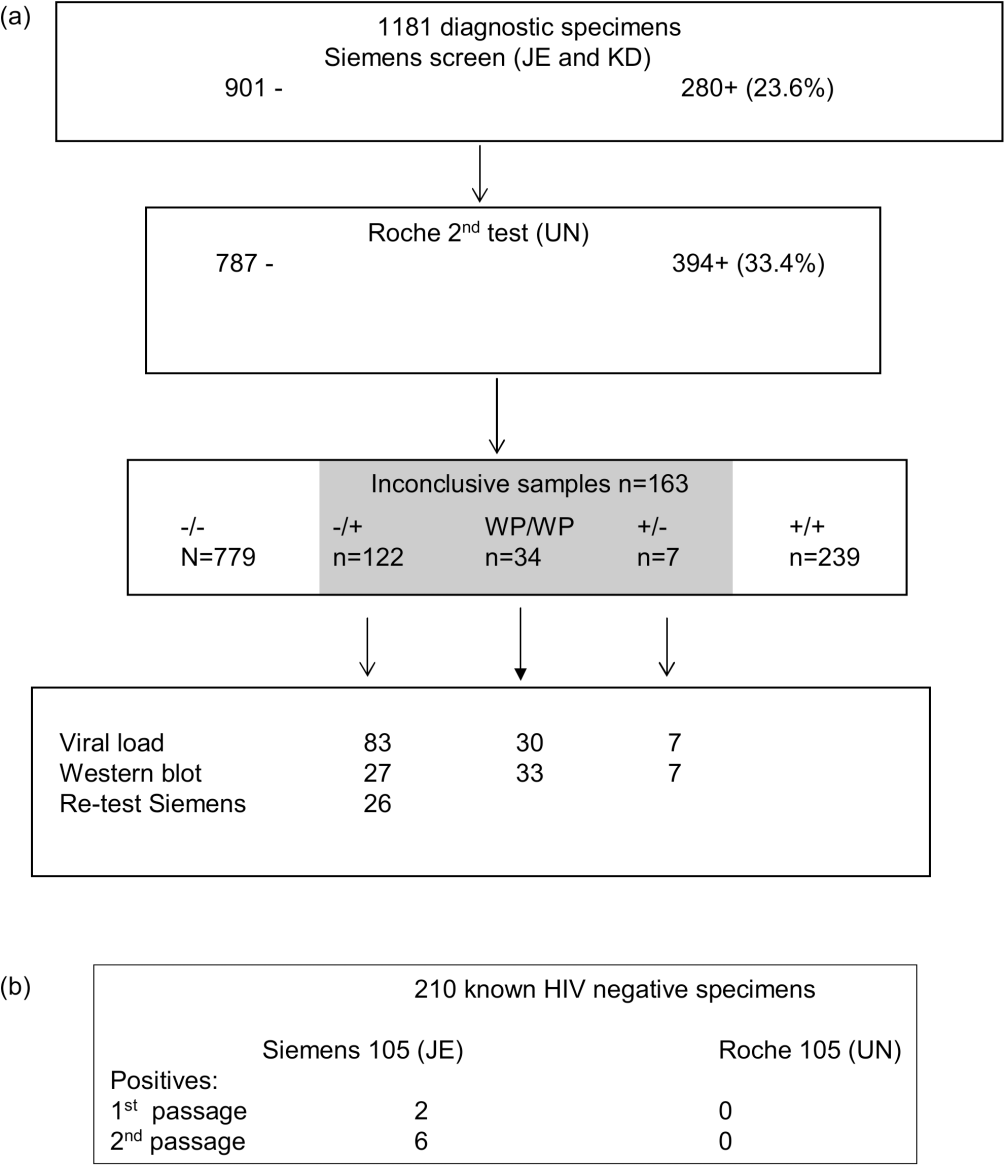
Study plan. (a) 1181 consecutive routine diagnostic samples (serum or plasma) tested on the Siemens HIV 4^th^ generation platform at the primary testing laboratories were re-tested on the Roche platform at the referral laboratory UN (using the same primary tube). Inconclusive specimens were further investigated with HIV viral load and western blot where specimen volume permitted. A subset of 26 specimens which were screen negative, second test positive was retested on the Siemens (initial screening assay) at the JE laboratory. (b) 210 known HIV antibody negative, NAT negative specimens from the same donor were tested on the Siemens and Roche platforms at JE and UN respectively. 105 specimens were tested on each analyser over a 10 day period and handled as normal clinical specimens. After the initial HIV serology test, the specimens were sent through the same analyser and tested for a second time.

This was a prospective study to evaluate the performance of two automated serology platforms through co-testing of routine specimens in three different laboratories. Routine diagnostic specimens from two medium sized public sector laboratories (KD and JE) were tested on the ADVIA Centaur 4^th^ generation HIV Ag/Ab combo assay (Siemens Healthcare Diagnostics, Deerfield, USA) over a 6 week period (April to May 2016). Irrespective of the result, specimens were referred to laboratory (UN) for blind repeat testing on the COBAS HIV Ag/Ab combo assay (Roche Diagnostics, Penzberg, Germany). (Fig 1) In all three laboratories, analysers were located in clinical pathology environments and used to perform routine chemistry as well as viral serology. Specimens were barcode labelled, uncapped and run through the analysers in their primary tubes where possible. Low volume specimens were poured into cups for testing.

### HIV viral load and western blot testing

Where residual specimen was available, HIV viral load and western blot were performed on inconclusive specimens to determine their true infection status. For HIV viral load and western blot the COBAS® AmpliPrep/COBAS® TaqMan® HIV-1 Test, v2.0, (Roche, Pensberg, Germany) and Genetic Systems ^TM^, HIV 1 Western Blot, Bio-Rad, Hercules, California, USA was used.

Most of the diagnostic specimens were serum. While plasma is the recommended matrix for the viral load assay, an in house validation was performed to confirm that both serum and plasma yielded equivalent viral load values. In addition, use of serum as a matrix for viral load testing has been used in previously published studies. [7]

In addition, laboratory records were reviewed and results of follow up HIV testing was recorded.

### Identification of false positive specimens

Discordant and weakly positive HIV serology may occur during primary infection. At this time, viral loads are typically high (>4 log copies/ml) [8]. Specimens were identified as false positive if viral load and western blots were both negative, or if follow up serology was negative.

### Investigation of first test negative/2^nd^ test positive specimens

To investigate whether an observed increase in assay positivity during the testing process was due to contamination with HIV positive serum, a subset of first test negative, second test positive specimens were retested on the Siemens platform at JE laboratory.

### Testing of known negative specimens

210 plasma specimens were prepared from a single HIV sero-negative, nucleic acid test (NAT) negative donor. 105 were tested on the Siemens analyser at JE laboratory and 105 on the Roche analyser at UN. Specimens were sent through the laboratories over a 10 day period and handled as normal clinical specimens. After the initial HIV serology test, the specimens were sent through the same analyser and tested for a second time. The results from the first and second testing episodes were compared.

### Experiment to detect antibody contamination of analyser surfaces

A Roche COBAS analyser in use in a clinical laboratory was used for this experiment. Surfaces were swabbed as follows: the instrument was opened up, a cotton swab was dipped in distilled water, and 25 locations inside or on the instrument were swabbed. The swab was placed in 500ul HIV-negative serum and vortexed. The swab was removed, the serum centrifuged, and the top 300ul serum was sent for HIV testing on the COBAS. Based on results, 10 of these sera were selected for HIV RNA detection, and one additional swab was taken in the same manner and serum used for Hepatitis B (HBV) DNA detection.

Negative control swabs were also tested. These included unused swabs in negative serum (n = 3) swabs of surfaces not exposed to the area in or around the instrument where contamination is believed to occur—specifically bench tops in the HIV PCR, HIV viral load, and non-automated serology laboratories, and the tea room tables (n = 4).

These were placed in negative serum and treated as described.

## Results

### HIV sero-positivity rises over first and second testing events

Altogether, 1181 consecutive clinical specimens (941 from KD and 240 from JE) were tested. Using the manufacturer’s cut off, 280 (23.7%) specimens tested positive and 901 (76.3%) tested negative on Siemens analysers.

**Fig 2:**
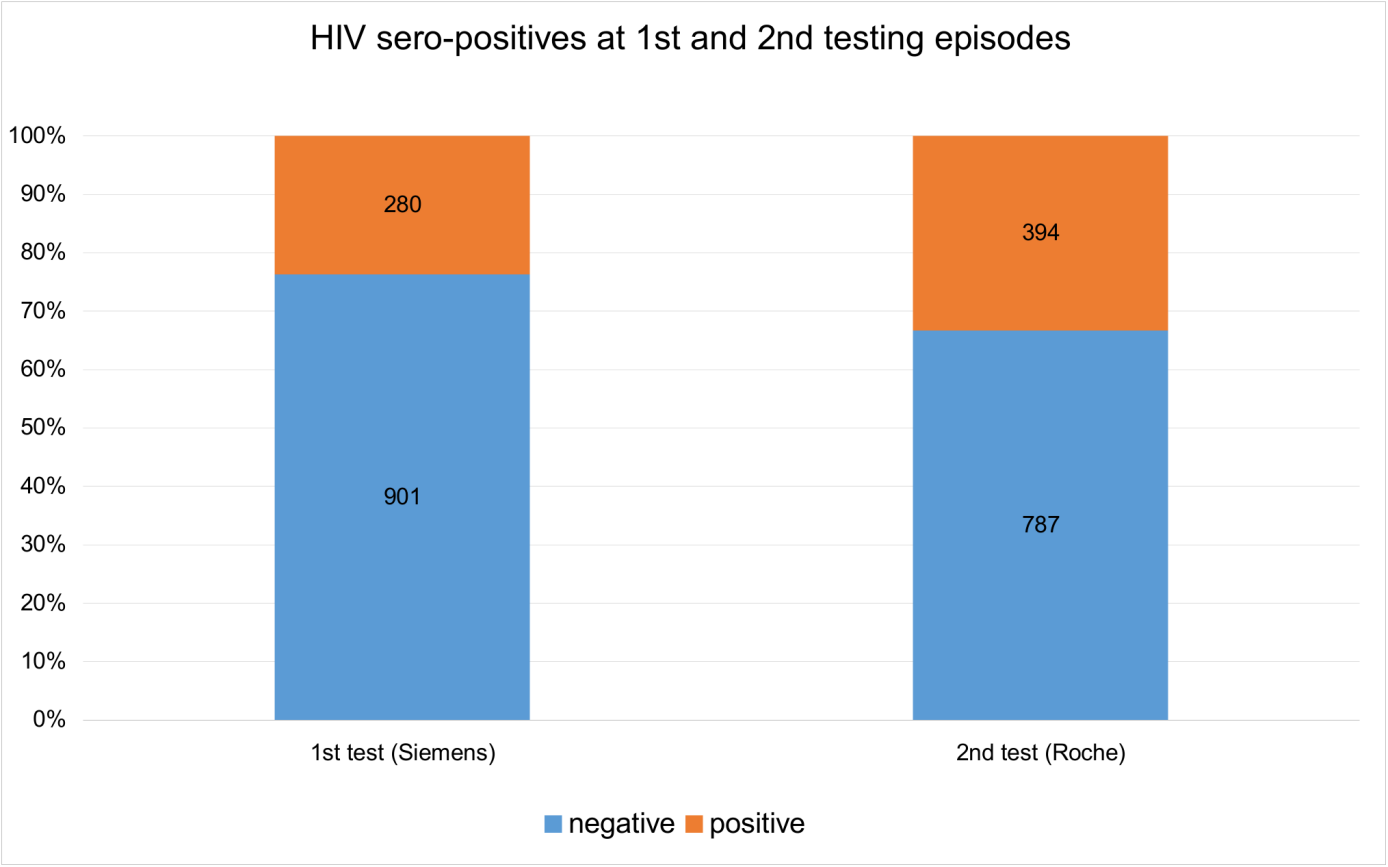
Determining sero-positivity at first and second testing episodes. The number of negative results decreased and the number of positive results increased on second testing on the Roche analyser at UN laboratory.

With second testing at laboratory UN on the Roche platform, the number of positive specimens increased substantially to 394 (33.4%). (Fig 2)

**Fig 3:**
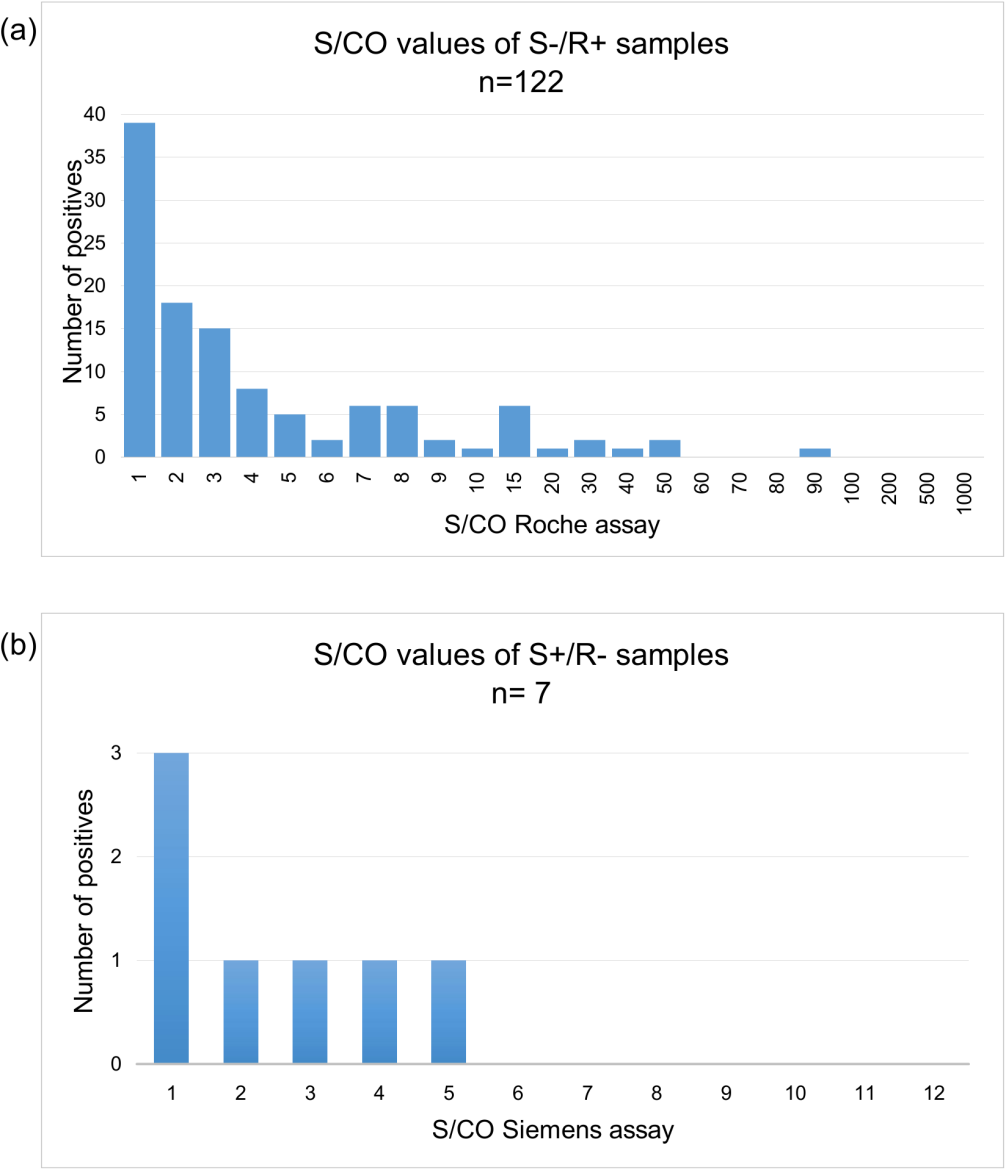
S/CO values of the positive test in S+/R- and S-/R+ discordant samples were generally close to the cut-off (weakly reactive). (a) S-/R+ samples had S/CO values between 1.0 and 99.1. (The positive range of Roche assay is 1->1000); (b) S+/R- samples had S/CO values between 1.1 and 5.8 (the positive range of Siemens assay is 1->12).

All of the discordant positives were weakly reactive with values <100 on Roche assay (range 1.0–99.12) or, <6 on the Siemens assay (range 1.1–5.8) (Fig 3A and 3B). In total 129 specimens of 1181 (13.8%) had discordant HIV status. Seven were first test positive, but 2^nd^ test negative (S+/R-) and 122 were first test negative, but 2^nd^ test positive. In addition, a further 34 samples were weakly positive in both assays (with values close to the cut offs for both assays).

### Viral load and western blot testing

To determine the true infection status of inconclusive samples, viral loads were performed on 91 discordant specimens which had sufficient volume for testing. HIV RNA was detectable in only 2 of 91. In both, the viral load was low, with levels below 100 copies per ml.

Viral loads were also done on 30 samples which were weakly positive in both HIV serology assays. Viral loads on three of these samples were high, compatible with HIV infection.

HIV-1 western blots were performed on 34 discordant specimens (7 S+ / R-, 27/122 S-/R+) as well as on 33 concordantly weakly positive specimens.

**Fig 4:**
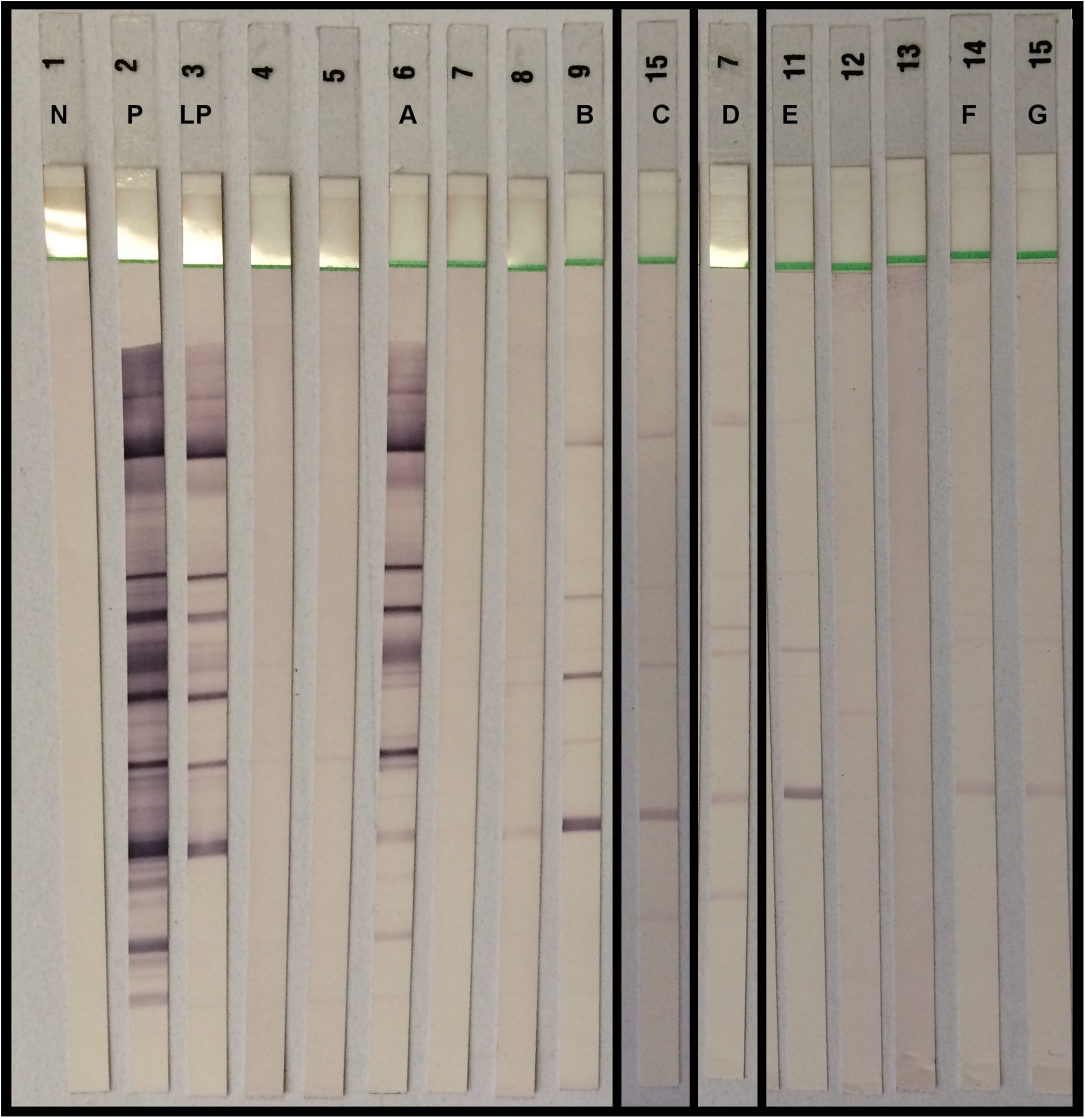
Western blots of selected specimens: negative, positive and low positive controls are labelled N, P and LP respectively. Specimens of interest are labelled A to G. Specimens A, B and C were concordantly weakly positive on both serology assays and HIV viral loads were high. Their western blots were scored as positive (A) and indeterminate (B, C). Specimens D, E, F and G were also WB indeterminate, but RNA negative. A follow up specimen on D was antibody negative.

Forty two samples were western blot negative. One specimen was scored positive (KD902, Table 1; specimen A, Fig 4). A further 24 showed weak reactivity with one or more HIV1 proteins and were scored indeterminate according to kit instructions (Fig 4). Two specimens (JE562 and KD136) were strongly HIV RNA positive (both primary HIV infections) (Fig 4, samples B and C). Weak reactivity in the western blots of RNA negative specimens is most likely due to trace amounts of contaminating HIV antibody. This was confirmed in sample D, when a follow up specimen was HIV antibody negative.

**Table 1:** Characteristics of the RNA positive samples.

Sample	Siemens S/CO	Roche S/CO	Viral load (c/mL)	Western blot	Follow up testing
JE562	5.18	45.24	124901	Indeterminate*: +gp160, +p55, +gp41, +p40, +p24, +p18	Strongly positive serology
KD902	7.7	76.36	5687	Positive: ++gp160, +++gp120, +++p65, ++p55, ++gp41, +p40, ++p31, +p24, +p18	Viral load 5558
KD136	6.04	95.63	533388	Indeterminate*: +gp160, +p55, +gp41, +p40, +p31, +p24	Strongly positive serology
KD073	7.03	26.56	136	Indeterminate*: +gp160, +p55, +p40, +p24	Viral load LDL
KD795	1.23	5.82	<100	Indeterminate*: +gp41	ND
KD527	2.61	13.1	<100	Negative	ND
KD998	0.96	7.65	<100	Negative	ND

*Faint bands present resulting in an indeterminate classification, as per manufacturer instructions.

### Investigation of first test (Siemens) negative/second test (Roche) positive specimens

The commonest inconclusive result were those that were S-/R+. Possible explanations could be: low sensitivity of the Siemens assay, greater non-specificity of the Roche assay, or more contamination because specimens were tested on the Roche analyser second. To investigate this, 26 S-/R+ specimens with sufficient volume were retested on the Siemens analyser at JE laboratory. On retesting, the S/CO increased in 25 of 26 specimens. Eleven specimens which had been negative became positive. Positive values ranged from 1.0 to >12 (Table 1S). The only explanation for this is that samples had become contaminated with HIV antibody during the testing process.

### Follow up records

Follow up results were available on 38 patients with inconclusive serology (32 HIV serology and 6 viral loads). In all instances follow up results confirmed the status we attributed to the study sample: 30 were HIV antibody negative and 2 strongly positive. The latter two were from patients JE562 and KD136, (Table 1). Five viral load follow up samples were lower than detectable limits (LDL) and one (KD902 (Table 1).) 5558 copies/ml. Taking the laboratory investigations and available follow up HIV testing into consideration, only 3 inconclusive sample were considered true positive and 47 were confirmed to be false positive (negative western blot and HIV viral load or negative follow up serology). It was not possible to confirm conclusively the status of the rest, due to the lack of follow up samples.

### Testing of known negative samples on both platforms

To provide more convincing evidence that sample contamination was responsible for the discordant results, specimens from the same HIV antibody, NAT negative donor were tested on the Roche and Siemens analysers. All 105 samples tested on the Roche analyser at UN were negative on first and second passage through the analyser. However, on the Siemens analyser at JE laboratory, 2 of 105 samples were positive on first passage through the analyser and 6 on second testing. Six of the 8 false positives remained positive on testing for a third time. (Table 2) Three of the 8 false positives occurred on the same day, suggesting a possible contaminating event could have occurred on that day. Of concern, one sample was repeatedly positive with a S/CO value >12. Thus the assay specificity dropped from 98.1% to 94.3% between first and second testing. This specificity is significantly lower than that claimed by the manufacturers.

**Table 2:** S/CO values of false positive samples from Siemens analyser at JE laboratory.

Sample ID	1st test	2nd test	3rd test
S003	1.08	0.06	
S022	<0.05	1.81	1.61
S030	<0.05	1.38	1.36
S031	0.07	4.37	3.86
S032	0.08	4.28	3.46
S033	0.09	1.50	1.72
S074	2.63	0.14	
S092	<0.05	>12	>12

### Evidence of serum contamination of internal and external surfaces of Roche analyser

10/25 (40%) of the swabs collected from internal and external surfaces of the analyser tested positive on the HIV serology assay, with S/CO ranging from 1.18 to 136.8. All control swabs of bench top surfaces from other parts of the laboratory were negative. This is compatible with contamination of the instrument surface with HIV positive serum and hence evidence that sample splashes can occur during the testing process. None of the antibody positive swab samples tested positive for HIV RNA. However, the swab tested for HBV DNA was positive. Contaminated areas included the sample loading bay, the chemistry module, and the internal sample transport system.

## Discussion

There is little data on the performance of automated serology platforms in high HIV prevalence settings. Both the ADVIA Centaur and the Roche 4^th^ generation HIV Combo assays are sensitive and specific and have excellent reliability in low prevalence settings [9], [10], [11], [12] where the priority is to detect HIV infection as early as possible. Assays are thus designed to detect minute amounts of HIV antigen or antibody, and nucleic acid testing is performed if serology is inconclusive [13]. In resource limited settings, reactivity in 2 HIV immunoassays (and in a follow up sample, if tested in a laboratory) is used to confirm infection [4]. Assay performance in routine clinical pathology settings has not been extensively tested in regions of high HIV sero-prevalence. This study was initiated to investigate a problem of non-specificity encountered in clinical pathology laboratories in South Africa. Weakly positive 4th generation HIV results were common and most were probably false positive: HIV RNA was rarely present at levels compatible with primary infection and follow up samples (available on 38 patients) were either antibody or viral load negative. In all, based on information available to us, 47 samples were conclusively shown to be false positive. Only 3 patients were shown conclusively to be HIV infected. These were probably early infections as viral loads were high, serology was weak on both platforms, western blots were positive or indeterminate and HIV serology on follow up was strongly positive. It is possible that there were additional true HIV infections amongst the inconclusive samples: virus replication is responsible for boosting antibody and levels can decline in elite controllers and patients on prolonged ART. [14], [15],[16] Such patients might have undetectable viral loads and low HIV antibody. However, this is rare and unlikely to account for the large number of weakly positive results we encountered.

The most common problem results were negative on the screening assay, but weakly positive on the second test. The most likely explanation is that samples were contaminated with HIV positive serum during the analytical process. Lines of evidence that support this interpretation include: 1) when Siemens negative/Roche positive samples were retested on the Siemens platform, the S/CO values increased, and up to half became positive. 2) Western blots of many HIV RNA negative samples showed evidence of low levels of HIV antibody (weak reactivity with one or more HIV bands). 3) Follow up samples on 35 patients were HIV antibody or RNA negative. 4) Eight known HIV negative specimens became HIV sero-positive on passage through the Siemens analyser at JE laboratory and six remained positive on repeated testing.

In a separate experiment to test the sensitivity of the Roche assay, a strongly positive sample was serially diluted. At a dilution of 10^−5^, the sample still tested positive with a S/CO of 6, proving that contamination with minute amounts HIV antibody can cause positivity. Bearing in mind that 20–30% of hospital/clinic-attending patients in South Africa are HIV positive, it is easy to envisage contamination occasionally happening during analysis. To test this, known negative samples from the same donor were repeatedly tested on both Siemens and Roche platforms. While no positives were detected in this experiment on the Roche analyser, an increasing number of positive events was observed during first and second passage on the Siemens analyser in JE laboratory. Of concern, one sample was repeatedly positive with a value of >12.

Sample contamination is a stochastic event and probably happens due to aerosols accidentally generated during the testing process. Evidence that sample splashes do indeed occur during the testing process came from swabbing experiments: 10 of 25 swabs from the surface and insides of a COBAS analyser in use in a clinical pathology laboratory tested positive for HIV antibody, with S/CO values ranging from 1.18 to 136.8. High risk areas were the sample loading bay, the chemistry module, and the internal sample transport system. Shortcuts with maintenance and prior passage of specimens through the chemistry module could exacerbate this. Serology modules use a separate tip for each sample, but the same probe enters each sample on most chemistry analysers.

Contamination may not only occur on the analyser, other pre and post analytic activities that could generate aerosols and cause contamination to surrounding samples include: de-capping the tubes, manual pipetting, agitation as samples are moved around the laboratory. The apparently poorer performance of the Roche analyser in the first experiment could have been due to the fact that the specimens were tested on this platform second. Some of the S-/R+ specimens may already have been contaminated prior to loading. No contaminating events were detected during the second experiment on the analyser at UN laboratory.

Sample contamination with HIV antibody was not the only cause of false positive results. Seven samples were first test positive on the Siemens, but negative on the Roche second test, HIV RNA and western blot. Also, not all S-/R+ specimens were Siemens positive on re-test (Table 1S). In a review on the causes of false positive HIV serology, Klarkowski et al [6] identified polyclonal B cell activation as the commonest cause of biological false positive results. Low avidity polyclonal antibody can cross react with antigens in one assay, but not in another and this is the basis for the use of an alternative HIV immunoassay to confirm a screening positive result [4].

This study highlights problems that can occur when HIV serology is performed in clinical pathology laboratories on general chemistry analysers, especially when they are used in a setting of high HIV prevalence. Contamination can easily occur and results must be carefully scrutinised before release. Contamination control measures need to be improved and workflow modified to ensure integrity of the specimen. To limit this problem, instrument cut offs should be adjusted based on local gray zone data, HIV serology testing should be prioritised on samples where chemistry tests are also needed. Ideally an HIV diagnosis should not be based on serology alone, but, in cases of doubt, confirmed with viral load and follow up testing.

## Supporting information

S1 Table Siemens screen and retest levels compared on a subset of 26 Siemens negative/Roche positive (S-/R+) samples. (DOCX)
