# Supplementary material for: Contamination with HIV antibody may be responsible for false positive results in specimens tested on automated platforms running HIV 4th generation assays in a region of high HIV prevalence
Source: PLoS One. 2017 Jul 31;12(7):e0182167. doi: 10.1371/journal.pone.0182167 (PMC5536287; doi:10.1371/journal.pone.0182167)
Supplement: S1 Table — (DOCX) [file pone.0182167.s001.docx]

**Table 1S Siemens initial and retest levels compared***

| **Roche (2^nd^ test)** | | **Siemens screen** | | **Siemens retest** |
| --- | --- | --- | --- | --- |
| 0.93 | <0.05 | | | 0.22 |
| 1.33 | 0.32 | | 0.35 | |
| 1.35 | 0.11 | | 0.22 | |
| 1.55 | 0.2 | | 0.42 | |
| 1.6 | 0.68 | | 0.63 | |
| 1.81 | 0.11 | | 0.56 | |
| 1.87 | 0.08 | | 0.08 | |
| 2.01 | 0.05 | | 0.47 | |
| 2.15 | 0.24 | | 0.900 | |
| 2.3 | 0.36 | | 0.69 | |
| 3.01 | 0.07 | | 0.56 | |
| 3.33 | 0.19 | | 0.84 | |
| 3.69 | 0.05 | | 1.23 | |
| 4.19 | 0.95 | | 1.06 | |
| 4.42 | 0.18 | | 2.51 | |
| 4.79 | 0.07 | | 0.51 | |
| 5.41 | 0.81 | | 0.90 | |
| 6.19 | 0.08 | | 0.92 | |
| 7.65 | 0.96 | | 1.21 | |
| 8.58 | 0.12 | | 8.25 | |
| 14.42 | 0.31 | | 2.80 | |
| 17.56 | 0.1 | | 4.48 | |
| 20.97 | 0.19 | | 3.13 | |
| 28.14 | 0.06 | | 4.24 | |
| 35.51 | 0.62 | | >12 | |
| 37.91 | 0.22 | | 3.69 | |

*Screen negative, second test positive samples were retested on the initial screening assay.
